# In planta localisation patterns of MADS domain proteins during floral development in *Arabidopsis thaliana*

**DOI:** 10.1186/1471-2229-9-5

**Published:** 2009-01-12

**Authors:** Susan L Urbanus, Stefan de Folter, Anna V Shchennikova, Kerstin Kaufmann, Richard GH Immink, Gerco C Angenent

**Affiliations:** 1 Plant Research International, Bornsesteeg 65, 6708 PD Wageningen, The Netherlands; 2 Laboratory of Molecular Biology, Wageningen University, Dreijenlaan 3, 6703 HA Wageningen, The Netherlands; 3 National Laboratory of Genomics for Biodiversity (Langebio), CINVESTAV-IPN, Campus Guanajuato, Apartado Postal 629, 36500 Irapuato, Guanajuato, Mexico; 4 Center "Bioengineering" RAS, prospect 60-letia Oktyabrya, 7, korp.1, 117312 Moscow, Russia; 5 Centre for BioSystems Genomics (CBSG), PO BOX 98, 6700 AB Wageningen, The Netherlands

## Abstract

**Background:**

MADS domain transcription factors play important roles in various developmental processes in flowering plants. Members of this family play a prominent role in the transition to flowering and the specification of floral organ identity. Several studies reported mRNA expression patterns of the genes encoding these MADS domain proteins, however, these studies do not provide the necessary information on the temporal and spatial localisation of the proteins. We have made GREEN FLUORESCENT PROTEIN (GFP) translational fusions with the four MADS domain proteins SEPALLATA3, AGAMOUS, FRUITFULL and APETALA1 from the model plant *Arabidopsis thaliana *and analysed the protein localisation patterns in living plant tissues by confocal laser scanning microscopy (CLSM).

**Results:**

We unravelled the protein localisation patterns of the four MADS domain proteins at a cellular and subcellular level in inflorescence and floral meristems, during development of the early flower bud stages, and during further differentiation of the floral organs. The protein localisation patterns revealed a few deviations from known mRNA expression patterns, suggesting a non-cell autonomous action of these factors or alternative control mechanisms. In addition, we observed a change in the subcellular localisation of SEPALLATA3 from a predominantly nuclear localisation to a more cytoplasmic localisation, occurring specifically during petal and stamen development. Furthermore, we show that the down-regulation of the homeodomain transcription factor *WUSCHEL *in ovular tissues is preceded by the occurrence of both AGAMOUS and SEPALLATA3 proteins, supporting the hypothesis that both proteins together suppress *WUSCHEL *expression in the ovule.

**Conclusion:**

This approach provides a highly detailed *in situ *map of MADS domain protein presence during early and later stages of floral development. The subcellular localisation of the transcription factors in the cytoplasm, as observed at certain stages during development, points to mechanisms other than transcriptional control. Together this information is essential to understand the role of these proteins in the regulatory processes that drive floral development and leads to new hypotheses.

## Background

Major developmental steps in flowering plants, such as the transition to flowering and floral organ development are, for the most part, controlled by members of the MADS domain family of transcription factors [1]. The action of these transcription factors in defining the identity of the floral organs has been captured in a genetic model, the "ABC" model [2], which was later extended with a "D" and "E" function [3-6]. This model describes how the combinatorial activity of several classes of regulatory genes, most of which encode MIKC-type MADS domain proteins, define the identity of the five different floral organs (sepals, petals, stamen, carpels, and ovules). According to this model, the combination of the class A+E genes specifies the identity of sepals, while the A+B+E genes specify petal identity, the combination of classes B+C+E determines stamen identity, C+E genes together lead to carpel identity, and finally the combination of classes C+D+E is responsible for ovule identity (for review see [7]). Floral organ development in *Arabidopsis thaliana *is controlled by the following genes: the A-function is represented by the genes *APETALA1 *(*AP1*) and *APETALA2 *(*AP2*) (not a MADS domain transcription factor); the B-function is controlled by *APETALA3 *(*AP3*) and *PISTILLATA *(*PI*); *AGAMOUS *(*AG*) represents the C-function; the D-function is represented in a redundant manner by *SEEDSTICK *(*STK*), *SHATTERPROOF1 *(*SHP1*) and *SHATTERPROOF2 *(*SHP2*); and the E-function involves the four closely related genes *SEPALLATA1 *to *SEPALLATA4 *(*SEP1-4*). Furthermore, another MADS box gene *FRUITFULL *(*FUL*), which is not described in the "ABC" model, is also involved in carpel development. In addition to these functions in floral organ development, some of these genes also have other functions. For instance, both *FUL *and *AP1 *are involved in the transition from inflorescence meristem to floral meristem identity, while *AG *controls the floral meristem determinacy [8-10].

The MADS domain proteins and the "ABC" model are well studied subjects for transcription factor regulation and action. Several studies have shown that at least some MADS domain proteins need to be in a dimeric form, either homo- or heterodimeric, before they can enter the nucleus [11-13]. In the nucleus, the proteins bind to DNA sequences of the target gene with the consensus CC(AT)_6_GG sequence, also known as the CArG box [14]. Binding to the DNA occurs either in the form of a dimer [14] or in a multimeric fashion [15,16], for instance in a tetrameric form as proposed in the "quartet" model [17]. Our knowledge about these MADS domain protein interactions has been greatly extended by a study where interaction data obtained from a systematic Yeast Two-Hybrid experiment was combined with large scale microarray co-expression data of the corresponding genes [18]. By considering not only the capacity of proteins to interact with each other, but also the possibility for putative partners to be co-localised in the same tissues and cells, the output is narrowed down to interactions that are likely to be of biological relevance for the plant.

However, microarray studies give only a very broad view on the spatio-temporal expression pattern of genes and do not provide the necessary detail that is needed to demonstrate co-localisation of the encoded proteins. In situ mRNA hybridisation studies and promoter-reporter studies like the ones reported for *AG *[19], *SEP3 *[20], *AP1 *[21], and *FUL *[22] reveal the expression patterns in more detail, but these might not reflect the protein localisation patterns. In fact, it is difficult to infer the protein localisation pattern from an mRNA expression pattern for two main reasons. First of all, production and degradation rates of mRNA and proteins could be totally different, and secondly, proteins can be transported from an expressing cell to a neighbouring non-expressing cell; all resulting in protein patterns that deviate from the mRNA patterns. It is known from studies on the class B genes *DEFICIENS *and *GLOBOSA *from *Antirrhinum majus *[23] that particular MADS domain transcription factors are able to transfer from one cell to another, where they may have a non-cell-autonomous function. Therefore, to obtain information about the spatio-temporal control of these regulatory proteins, it is essential to study the localisation of the proteins themselves. Additionally, protein localisation studies can be more informative on the functioning of transcription factors by showing the specific subcellular localisation of the proteins during development. It has been demonstrated that some MADS domain transcription factors are localised in the cytoplasm when an interaction partner is absent and only become functional when they enter the nucleus after dimerisation [11-13,24]. Preferably, one would like to obtain a three-dimensional map of protein localisations with cellular resolution and information about the dynamics of proteins during plant development. The discovery of GREEN FLUORESCENT PROTEIN (GFP) and similar fluorescent proteins and their use as visual tags for proteins, in combination with Confocal Laser Scanning Microscopy (CLSM) has made this visualisation of fluorescently tagged proteins in living plant tissue possible [25-27].

In order to study MADS domain proteins in living tissues with CLSM during floral development, we made C-terminal GFP tagged versions of *SEP3, AG*, *FUL*, and *AP1*. Previously it was shown that the fusion of GFP to the C-terminus of the MADS domain protein AP1 does not affect its function, as it is able to complement the *ap1 *mutant [28]. Furthermore, it is known from studies with *AG*, *STK*, and *SEP3 *that introns can contain important regulatory elements that are required for the correct expression pattern of these MADS box genes [29-31]. For this reason, we made C-terminal GFP tagged versions of *SEP3*, *AG*, *FUL*, and *AP1 *using genomic fragments [29]. Here we describe the protein localisation patterns of *gSEP3:GFP, gAG:GFP*, *gFUL:GFP *and *gAP1:GFP *on a cellular and subcellular level in the inflorescence meristem and at various stages of floral meristem and organ formation. This detailed study reveals discrepancies between the previously reported mRNA expression patterns and the protein localisations, and sheds new light on the functioning of the MADS domain proteins in floral organ patterning and formation.

## Results and discussion

In this study we analysed the spatio-temporal protein localisation patterns of C-terminally GFP tagged genomic clones of MADS domain proteins SEP3, AG, FUL and AP1 during floral development, hereafter referred to as SEP3:GFP, AG:GFP, FUL:GFP, and AP1:GFP. The generated constructs were transformed to *Arabidopsis thaliana *wild type Col-0 plants, and at least four GFP-expressing stable primary transformants per construct were analysed for their protein localisation. These were found to be very similar in localisation patterns, although some differences in expression levels were observed. These differences in expression levels may be due to differences in transgene copy numbers, but they may also be caused by positional effects of the insertion of the transgene. Three constructs, namely AG:GFP, FUL:GFP and AP1:GFP, were also introduced into their respective mutant lines. These complementation experiments showed that C-terminal GFP tagged MADS domain proteins are functional, as the AG:GFP, FUL:GFP and AP1:GFP proteins can rescue the mutant phenotypes of *ag*, *fu1 *and *ap1 *mutants, respectively. As the single *sep3 *mutant shows very subtle phenotypic alterations due to the redundancy of SEP3 with SEP1 and SEP2 [4], the SEP3:GFP construct was only transformed into the wild type background. The spatio-temporal protein localisation patterns described here are from representative lines homozygous for the transgene in the Col-0 wild type background.

### SEP3 localisation in inflorescence meristem and early flower bud stages

**Figure 1:**
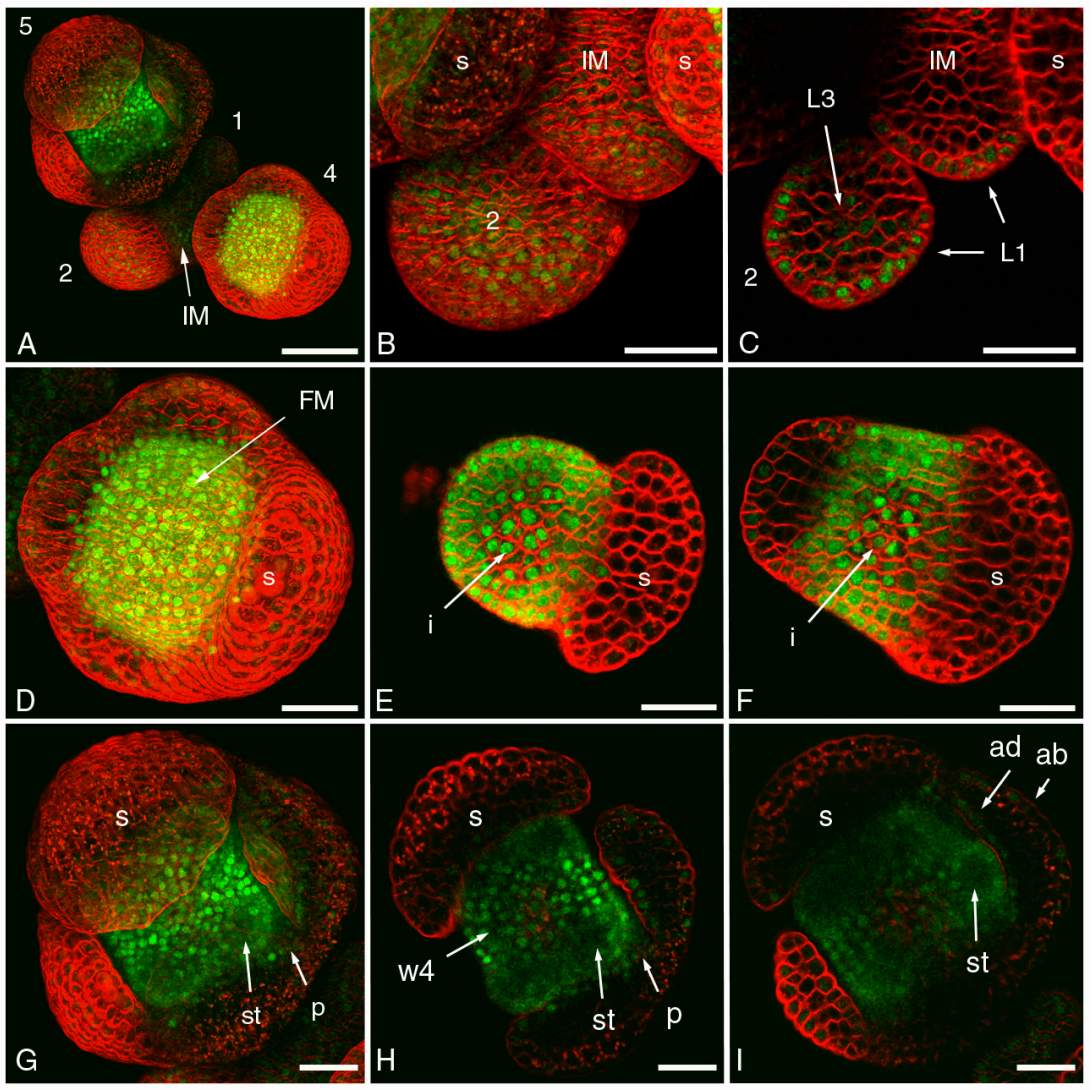
**Confocal microscopic analysis of SEP3:GFP localisation in inflorescence meristem and early flower bud stages**. (A) Overview of an inflorescence with the inflorescence meristem and early flower bud stages 1 to 5 indicated. SEP3:GFP protein is detected as green signal and cell membranes are stained with the red dye FM4-64. (B) Detail of an inflorescence meristem and a stage 2 flower bud. (C) Section through tissue in (B) showing the SEP3:GFP signal in the epidermis and the beginning SEP3:GFP signal in the centre of the stage 2 flower bud. (D) Detail of a stage 4 flower bud showing the highest SEP3:GFP signal in the entire floral meristem and only in the epidermis of the four sepals. (E) Section through the stage 4 flower bud in (D) showing both cytoplasmic and nuclear localisation of SEP3:GFP in the future second and third whorl, and only nuclear localisation in the innermost part of the floral meristem. (F) More basal section through the stage 4 flower bud in (D) showing again both cytoplasmic and nuclear localisation of SEP3:GFP in the future second and third whorl. (G) Detail of a stage 5 flower bud showing the initiating petal and stamen primordia. (H) Section through the stage 5 flower bud in (G) illustrating that the SEP3:GFP signal in whorl 4 is mostly nuclear, while the reduced signal in the initiating petal and stamen primordia is both cytoplasmically and nuclear localised. (I) More basal section through the stage 5 flower bud in (G) showing the SEP3:GFP signal at the adaxial and abaxial sides of a sepal. (1–5) flower bud stages; (ab) abaxial; (ad) adaxial; (FM) floral meristem; (IM) inflorescence meristem; (i) innermost part floral meristem; (L1) epidermal cell layer; (L3) inner cell layers; (p) petal; (s) sepal; (st) stamen; (w4) whorl 4. Scale bars of (A) 50 μm and of (B-I) 25 μm.

In the inflorescence meristem, SEP3:GFP plants had a very low but definite signal in the epidermal layer that was located mostly in the nucleus (Figures 1A–C). During the initiation of the floral primordia and flower bud stages 1 and 2 this epidermal localisation pattern remained. However, stage 2 flower buds also showed a few cells in the subepidermal and inner cell layers in the centre of the floral meristem that started to have a much higher level of SEP3:GFP signal, also located mostly in the nucleus (Figure 1C). During the development towards stage 3 flower buds this signal spread out to encompass the whole dome of the floral meristem from which the second, third and fourth whorl will develop at later stages (Figure 1D). This increasing SEP3:GFP localisation fits with the reported mRNA expression pattern [20], where *SEP3 *expression starts in late stage 2 flower buds and is largely confined to the three inner whorls of the flower primordium. However, the weak, but distinct epidermal presence of SEP3:GFP in the inflorescence meristem and stage 1 flower buds has not been reported before. This signal could originate from the epidermis itself, but it cannot be excluded that it is the result of epidermal transport from nearby, high expressing tissues, such as the floral meristem in stage 2/3 flower buds. We also observed signal in the epidermal layer of sepal primordia. During the development of the sepals, the SEP3:GFP signal became weaker at the abaxial side of the sepals, while the signal remained at the adaxial side (Figure 1I). In agreement with this, low level *SEP3 *expression is occasionally detected on the adaxial side of sepals at later stages [20], and it was shown that *SEP1*-*SEP4 *are involved in specifying adaxial sepal surface identity [6].

Interestingly, the subcellular localisation of the SEP3:GFP protein changed dramatically from stage 3 onwards. Just before the petal and stamen initiation, SEP3:GFP proteins in the future second and third whorl became both cytoplasmically and nuclear localised. At the same time, the proteins in the innermost part of the floral meristem clearly remained nuclear localised (Figures 1E and 1F). This cytoplasmic SEP3:GFP signal in the second and third whorl could be due to higher expression levels of the gene, resulting in a temporary accumulation of SEP3:GFP proteins in the cytoplasm waiting for transportation to the nucleus. Another option could be that the appearance of new interaction partners in this region results in the cytoplasmic localisation of SEP3:GFP. For instance, it is known that the expression of *AP3*, which determines petal and stamen identity together with *PI *and *SEP3*, also starts at stage 3 and is restricted to the same area where whorl 2 and 3 will develop [32]. It would be interesting to investigate if AP3 is also located predominantly in the cytoplasm at this point in development. Another possibility could be that higher cell division rates in the area of the future second and third whorl which cause the petal and stamen primordia to arise, result in increased unloading of previously nuclear localised proteins into the cytoplasm [33]. The smaller cell sizes in the area of the future second and third whorl compared to the innermost part of the floral meristem could indicate higher cell division rates (Figure 1E). This unloading of the nuclear localised proteins into the cytoplasm during cell division may also allow the proteins to meet new partners and form new complexes in the cytoplasm, for instance with AP3 and PI. In stage 5 flower buds, the initiating petal and stamen primordia revealed an epidermal layer with mostly nuclear SEP3:GFP, while the subepidermal and inner cell layers showed a lower SEP3:GFP signal, both cytoplasmically and nuclear localised (Figures 1G–I). Apparently, SEP3 is less needed in the inner layers of the emerging petals and stamen than in the epidermis. In the mean time the SEP3:GFP proteins in the fourth whorl remained nuclear localised (Figure 1H).

### AG localisation in inflorescence meristem and early flower bud stages

**Figure 2:**
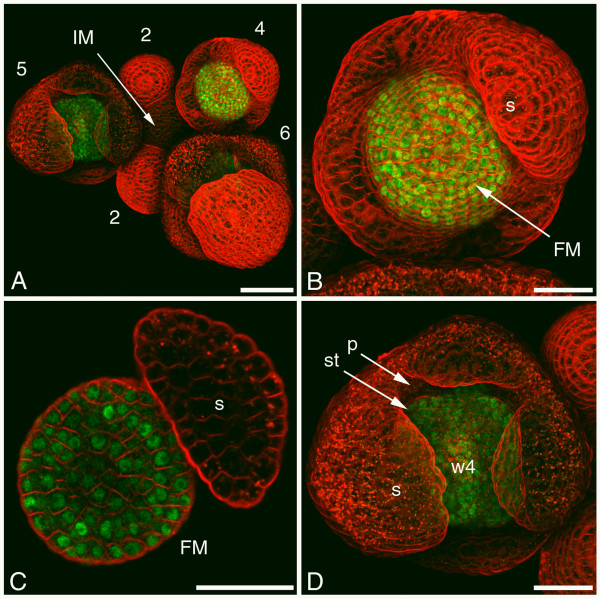
**Confocal microscopic analysis of AG:GFP localisation in inflorescence meristem and early flower bud stages**. (A) Overview of an inflorescence with the inflorescence meristem and early flower bud stages 2 to 6 indicated. AG:GFP protein is detected as green signal and cell membranes are stained with the red dye FM4-64. (B) Detail of a stage 4 flower bud showing AG:GFP signal in the future third and fourth whorl. (C) Section through the stage 4 flower bud in (B) showing that AG:GFP is located in both the cytoplasm and the nuclei. (D) Detail of the stage 5 flower bud in (A) showing AG:GFP signal in the developing stamen and in whorl 4. (2–6) flower bud stages; (FM) floral meristem; (IM) inflorescence meristem; (p) petal; (s) sepal; (st) stamen; (w4) whorl 4. Scale bar of (A) 50 μm and scale bars of (B-D) 25 μm.

The AG:GFP signal appeared in a cluster of subepidermal and inner layer cells in very early stage 3 flower buds, at the time when the first sepal primordium started to arise. During the development of stage 3 flower buds the AG:GFP localisation enlarged to encompass the part of the floral meristem from which the third and fourth whorl will develop, and this pattern remained in later stages (Figures 2A, B, and 2D). This corresponds well to the observed *AG *mRNA expression pattern that starts in the floral meristem of stage 3 flower buds and continues in whorl 3 and 4 in later stages [34,35]. During all the early stages of flower bud development the AG:GFP protein seemed to be primarily localised in the nucleus, but a substantial part of the signal was also localised in the cytoplasm (Figure 2C). In a stage 5 flower bud, AG:GFP was present throughout all cell layers of the developing stamen primordia and in the region of whorl 4 (Figure 2D).

### FUL localisation in inflorescence meristem and early flower bud stages

**Figure 3:**
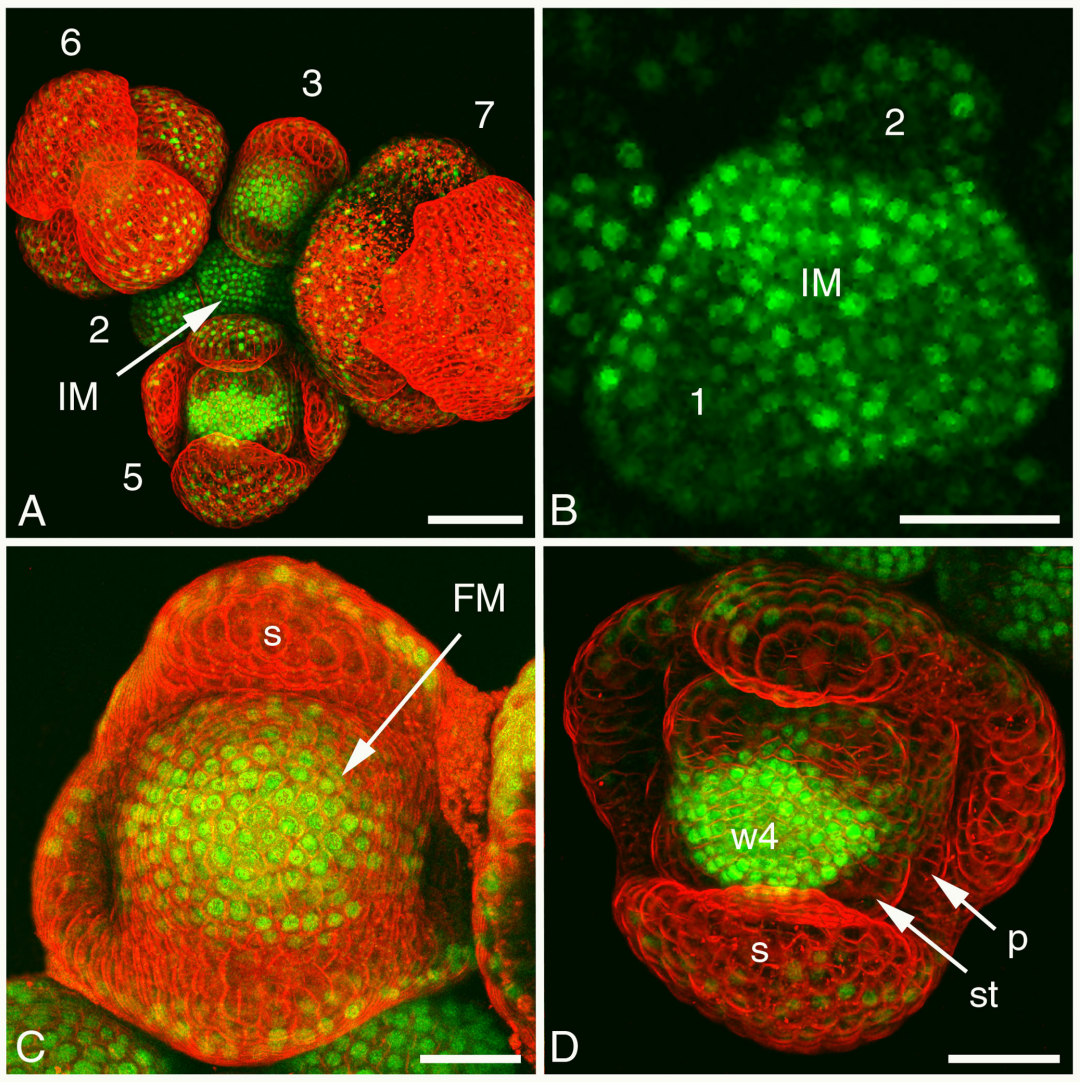
**Confocal microscopic analysis of FUL:GFP localisation in inflorescence meristem and early flower bud stages**. (A) Overview of an inflorescence with the inflorescence meristem and early flower bud stages 2 to 7 indicated. FUL:GFP protein is detected as green signal and cell membranes are stained with the red dye FM4-64. (B) Section through an inflorescence meristem showing high FUL:GFP signal in all layers, while stage 1 and 2 flower bud primordia have reduced signal. (C) Detail of a stage 3 flower bud showing the highest FUL:GFP signal in the floral meristem and only epidermal signal in the four sepals. (D) Detail of a stage 5 flower bud showing the highest FUL:GFP signal in whorl 4 and reducing signal in the petal and stamen primordia. (2–7) flower bud stages; (FM) floral meristem; (IM) inflorescence meristem; (p) petal; (s) sepal; (st) stamen; (w4) whorl 4. Scale bar of (A) 50 μm and scale bars of (B-D) 25 μm.

In the inflorescence meristem, FUL:GFP plants exhibited a very high fluorescence signal throughout all cell layers (Figure 3A). This signal was mostly located in the nucleus, but also in the cytoplasm (Figure 3B). As soon as the flower bud primordia were initiated the FUL:GFP signal started to reduce in the subepidermal and inner cell layers (Figure 3B), while in the centre of the emerging flower buds some cells maintained the high level of FUL:GFP signal. During the development of the sepals, the basal part of the sepal had FUL:GFP proteins in both epidermal and subepidermal layer, whereas the apical part of the sepal had only epidermal FUL:GFP. This FUL:GFP signal was maintained at the abaxial side of the sepal, whereas the adaxial side and the abaxial side at the tip showed a reduction in signal (Figures 3C and 3D). In stage 3, 4 and 5 flower buds the floral meristem had the highest amount of FUL:GFP protein in the innermost part of the floral meristem, while in the area of the future second and third whorl, the amount gradually reduced. However, in these initiating petal and stamen primordia the presence of FUL:GFP in the epidermis remained the longest (Figures 3C and 3D).

*FUL *mRNA is expressed in two distinct phases: in the inflorescence meristem and in the centre of the floral meristem from stage 3 flower bud onwards, but not in the intermediate flower bud stages 1 and 2 [22,36]. Therefore, the FUL:GFP presence that was observed in flower bud stages 1 and 2 (Figures 3A and 3B) might be due to FUL:GFP protein that remained from previous stages. The renewed *FUL *gene expression in stage 3 flower buds in the centre of the floral meristem corresponds with the increased FUL:GFP protein accumulation in the centre of the floral meristem from stage 3 onwards (Figure 3C). In summary, throughout the development from inflorescence meristem to floral organs it seems that the tissues that need to remain undifferentiated, such as the inflorescence and floral meristem, have FUL:GFP protein in all cell layers. Differentiating tissues however, like the developing floral organs, seem to loose the subepidermal and inner cell layer FUL:GFP signal and only retain the signal in the epidermis.

### AP1 localisation in inflorescence meristem and early flower bud stages

**Figure 4:**
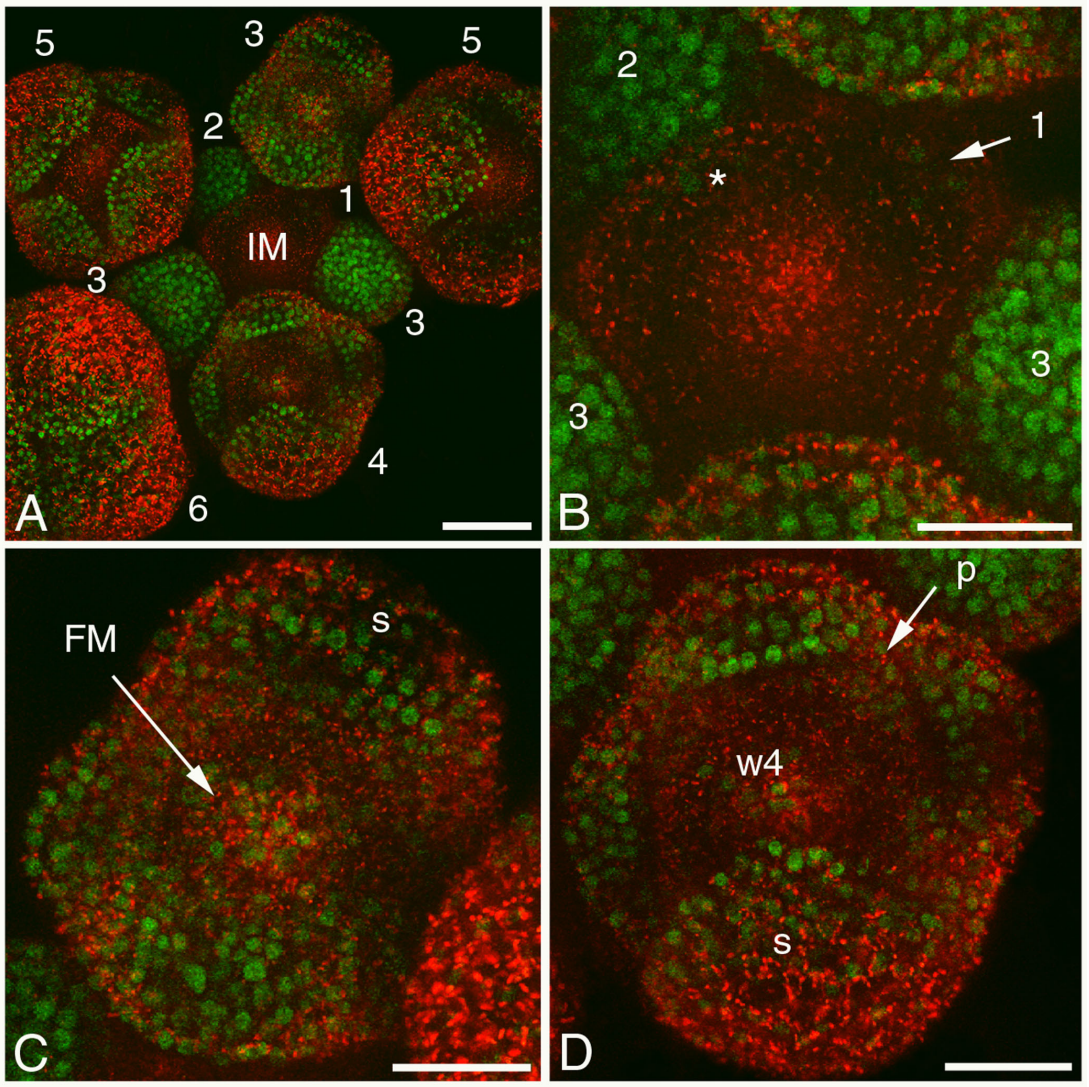
**Confocal microscopic analysis of AP1:GFP localisation in inflorescence meristem and early flower bud stages**. (A) Overview of an inflorescence with the inflorescence meristem and early flower bud stages 1 to 6 indicated. AP1:GFP protein is detected as green signal in a red autofluorescent background. (B) Detail of the inflorescence meristem in (A) showing that the AP1:GFP signal starts in a few cells in stage 1 flower buds and spreads to all cell layers in flower bud stage 2 and 3. The asterisk denotes AP1:GFP signal in nuclei of the adjacent stage 2 flower bud projecting through the overlying inflorescence meristem tissue. (C) Detail of a late stage 3 flower bud showing AP1:GFP signal in all cell layers of the four sepals and the floral meristem. (D) Detail of a stage 4 flower bud showing the highest AP1:GFP signal in the tips of the four sepals and lower signal at positions where the petals will emerge. The signal in whorl 3 and whorl 4 is reducing, although the signal does remain in whorl 4. (1–6) flower bud stages; (FM) floral meristem; (IM) inflorescence meristem; (p) petal; (s) sepal; (w4) whorl 4. Scale bar of (A) 50 μm and scale bars of (B-D) 25 μm.

The AP1:GFP signal was first detected in a few cells of the epidermal and internal cell layers of the emerging flower bud primordium (Figures 4A and 4B). From flower bud stages 1 to 3 the AP1 fusion protein was found throughout all cell layers and was located predominantly in the nucleus (Figure 4C). In flower bud stages 4 and 5, the AP1:GFP protein in the sepals was most abundant in the apical tips (Figures 4A and 4D). The signal diminished in the third and fourth whorl at this stage, while the signal remained in the second whorl where the petal primordia would emerge. However, a few epidermal cells in the centre of the fourth whorl still had a low AP1:GFP signal, perhaps representing the last meristematic cells in the differentiating floral meristem (Figure 4D). This localisation pattern corresponds with the reported *AP1 *mRNA expression [21,37], where *AP1 *expression starts in stage 1 flower bud primordia and increases during the development of the flower bud until stage 3. At the end of stage 3, the *AP1 *expression starts to reduce in the centre of the floral meristem as a result of the negative regulation of AG protein present there [37] (Figures 2A and 2B). However, as our AG:GFP and AP1:GFP localisation studies revealed, there is a clear time-lag between the termination of *AP1 *mRNA expression in early stage 3 flower buds, and the reduction in time of the AP1:GFP protein starting in late stage 3 flower buds (Figure 4C). The same time-lag is seen for the reduction of the FUL:GFP signal starting in stage 1 flower buds (Figure 3B), where *FUL *mRNA expression is suppressed due to the presence of AP1 protein [36]. This negative regulation of AP1 on *FUL *expression is probably also apparent in the developing sepals, where eventually the FUL:GFP protein presence is highest in the basal parts of the sepals (Figure 3D), while AP1:GFP protein is more abundant in the apical tips of the sepals (Figure 4D).

### SEP3, FUL and AP1 localisation during petal development

The function of the MADS domain proteins in the floral transition and in the determination of floral organ identities is well established, however, less is known about putative later functions of MADS domain proteins in differentiating floral organs. Recently, a report showed that *AG *has a late function in stamen development [38], and it was already known that *FUL *has a late function in pistil and fruit development [22,39]. In view of this and the fact that MADS domain proteins were still present in differentiating floral organs, we studied the SEP3:GFP, FUL:GFP and AP1:GFP localisations during the development of the petal in more detail.

**Figure 5:**
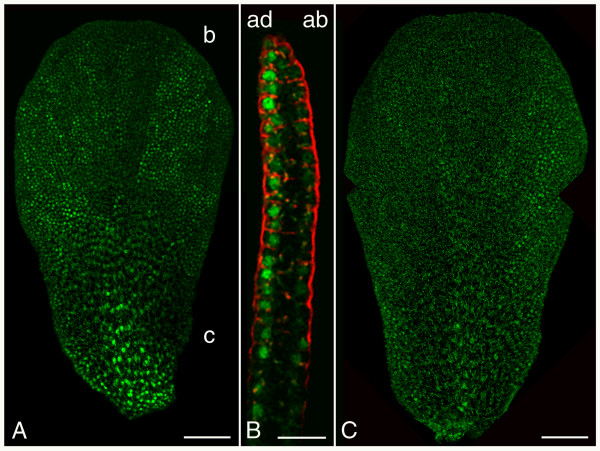
**Confocal microscopic analysis of SEP:GFP during petal development**. (A) SEP3:GFP signal at the adaxial side of a stage 11 petal. Nuclei with SEP3:GFP proteins are visible as bright green spots against the green autofluorescent background of the petal. (B) Cross section through a petal of approximately stage 10 showing higher SEP3:GFP signal in the adaxial epidermis than the abaxial epidermis. Cell membranes are stained with the red dye FM4-64. (C) SEP3:GFP signal at the abaxial side of a stage 11 petal. The images in (A) and (B) are each composed of two separate, overlapping projections. (ab) abaxial side; (ad) adaxial side; (b) blade of petal; (c) claw of petal. Scale bars of (A) and (C) 50 μm and scale bar of (B) 15 μm.

At stage 5, we observed that SEP3:GFP signal was predominantly located in the epidermis of the emerging petal primordium (Figures 1G and 1I), while AP1:GFP signal was present throughout all cell layers (Figure 4D). FUL:GFP protein was hardly present at this stage of petal development (Figure 3D). Around stage 9, when petals start to increase rapidly in size [40], high AP1:GFP signal was detected throughout all cell layers. This signal slowly reduced in time and was almost abolished in a stage 12 petal (data not shown). This is in agreement with the *AP1 *mRNA expression pattern reported for petals [21,37]. Remarkably, FUL:GFP protein was observed in the centre of the claw around stage 10 (data not shown), where it might be involved in the vascular development of the petal [22]. The SEP3:GFP signal in a stage 9 petal was higher in the adaxial epidermis of the petal than the abaxial epidermis (Figure 5B). This difference in adaxial and abaxial patterning of SEP3:GFP fusion protein remained until stage 12 petals, although the signal gradually reduced. In a stage 11 petal the SEP3:GFP signal on the adaxial side was strongest in the blade and in the middle of the claw (Figure 5A), while at the abaxial side the edges of the blade and the middle of the claw had the strongest SEP3:GFP signal (Figure 5C). The asymmetric accumulation of SEP3:GFP protein in the petal epidermis is in contrast with the uniform *SEP3 *mRNA expression reported for petals [20], but it does resemble the epidermal SEP3:GFP localisation pattern that we observed in sepals (Figure 1I), suggesting that SEP3 could play a role in the adaxial/abaxial patterning of both organs.

### FUL, SEP3 and AG localisation during pistil development

It is well-known that *FUL *has a late function in valve differentiation in the developing pistil and in fruit elongation [22,39]. In contrast, much less is known about putative late functions of *SEP3 *and *AG *during pistil development. Therefore, we studied the localisation patterns of FUL:GFP, SEP3:GFP and AG:GFP in the developing pistil until flower bud stage 12 (stage after [40]), when style, valves, valve margins, and replum are being formed. Note that in stage 12 pistil tissue the confocal microscope laser could not reliably penetrate beyond five cell layers.

**Figure 6:**
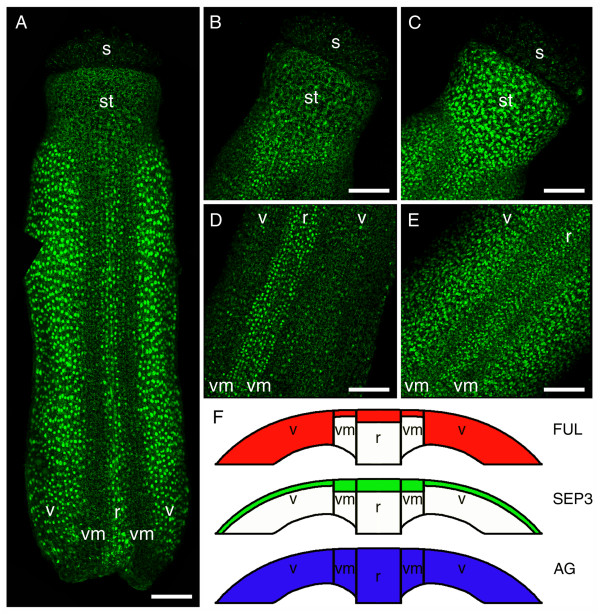
**Confocal microscopic analysis of FUL:GFP, SEP3:GFP and AG:GFP localisation in stage 12 pistils**. (A) FUL:GFP localisation in a stage 12 pistil, with the highest signal in the valves and the replum, and lower signal in the valve margins and the basal half of the style. The image is composed of three separate, overlapping projections. Nuclei with FUL:GFP proteins are visible as bright green spots against the green autofluorescent background of the pistil. (B) SEP3:GFP localisation in the apical part of a stage 12 pistil with the highest signal in the valve margins and replum, and lower signal in the style and valves. (C) AG:GFP signal in all cell layers of the apical part of a stage 12 pistil. (D) SEP3:GFP localisation in the middle part of a stage 12 pistil with the highest signal in the valve margins and replum, and lower signal in the valves. (E) AG:GFP signal in all cell layers of the middle part of a stage 12 pistil. (F) Schematic cross sections of the FUL:GFP, SEP3:GFP and AG:GFP localisations in the ovary wall. (r) replum; (s) stigma; (st) style; (v) valve; (vm) valve margin. All scale bars 50 μm.

We observed that FUL:GFP is predominantly located in the two valves and the replum and, to a much lesser extent, in the valve margins and the basal half of the style (Figure 6A). In the valves FUL:GFP was present in the first five cell layers, whereas we detected signal in the replum and the style only in the first two layers, and in the valve margins only in the first layer (Figure 6F). In a younger gynoecium (stage 10) this FUL:GFP localisation pattern was already visible, although with lower intensity, but in this stage the future replum had only relatively high signal in the basal part (data not shown). The presence of FUL in the epidermal and subepidermal layers of the replum and valve margins has not been reported before in either developing pistils or fruits [9,22,36]. Nevertheless, *ful-1 *mutant fruits do not only have defects in the valve tissue but also fail to dehisce, indicating defects in the replum and valve margins [9,22]. Also, the presence of the FUL:GFP protein in the replum does not transform the cells into valve cells, whereas this was reported happening when constitutively expressing *FUL *in the entire gynoecium [41]. This suggests that either the replum identity is already established prior to or around stage 10 before the FUL:GFP expression is basal-apically up-regulated in the replum, or that for the conversion of replum to valve cells FUL protein needs to be present in all cell layers of the replum. The suggestion that FUL could be intercellularly transported from the valve tissue to the replum [22] is supported by our localisation data. On the other hand, most studies on *FUL *expression focused more specifically on fruit development (after stage 12), therefore, it could be that *FUL *expression in the epidermal and subepidermal layers of the replum and valve margins in the developing pistil (until stage 12) has been overlooked.

SEP3:GFP fusion protein is most abundant in the replum and the valve margins, while the valves and the style showed a much lower signal (Figures 6B and 6D). In the replum and the valve margins SEP3:GFP was located in the first two cell layers, whereas the style and the valves had only signal in the epidermal layer at this stage of pistil development (Figure 6F). However, in a younger gynoecium (stage 10) the same localisation pattern with lower intensity was found, but at least three cell layers deep in the future style and two cell layers deep in the future valve margins, the future replum, and the future valves (data not shown). Therefore, it seems that during the development of the gynoecium SEP3:GFP becomes restricted to the epidermal and subepidermal layers.

We observed that AG:GFP is present throughout all cell layers of the whole pistil including the style and stigma (Figures 6C, E and 6F). In a younger gynoecium (stage 10) the same pattern is already present (data not shown). It was shown that *AG *mRNA expression at early gynoecium stages is throughout the whole tissue, but in contrast to our protein localisation data, this expression becomes restricted over time to the stigma and no or hardly any expression exists in the ovary walls in a stage 12 pistil [19]. The high uniform signal of AG:GFP that we observed throughout stage 10 and stage 12 pistils could be explained by a low turnover of the protein molecules in those tissues.

### SEP3 and AG localisation correlated with *WUS *expression during ovule development

We studied the spatio-temporal localisation pattern of SEP3:GFP and AG:GFP during ovule development in relation to the expression pattern of *WUSCHEL *(*WUS*). *WUS *is a homeodomain transcription factor involved in meristem cell identity maintenance in shoots and an important regulator of ovule development [42,43]. It is expressed in the centre of the shoot apical meristem, inflorescence and floral meristem and later in the developing ovule [43-45]. The down-regulation of *WUS *in the terminating floral meristem is thought to be regulated by AG [46,47]. Additionally, D- and E-type MADS domain proteins like STK and SEP3, might be involved in the suppression of *WUS *and the termination of the floral meristem [48] (RI and GA, unpublished results). In ovules, *WUS *is thought to be down-regulated by the SEP3-AG dimer combined with homeodomain transcription factor BELL1 [49]. For this reason, the localisation pattern of SEP3:GFP and AG:GFP was correlated with the expression pattern of *WUS *in ovules, which may reveal whether this interaction between these MADS domain proteins and the *WUS *gene is tightly correlated.

**Figure 7:**
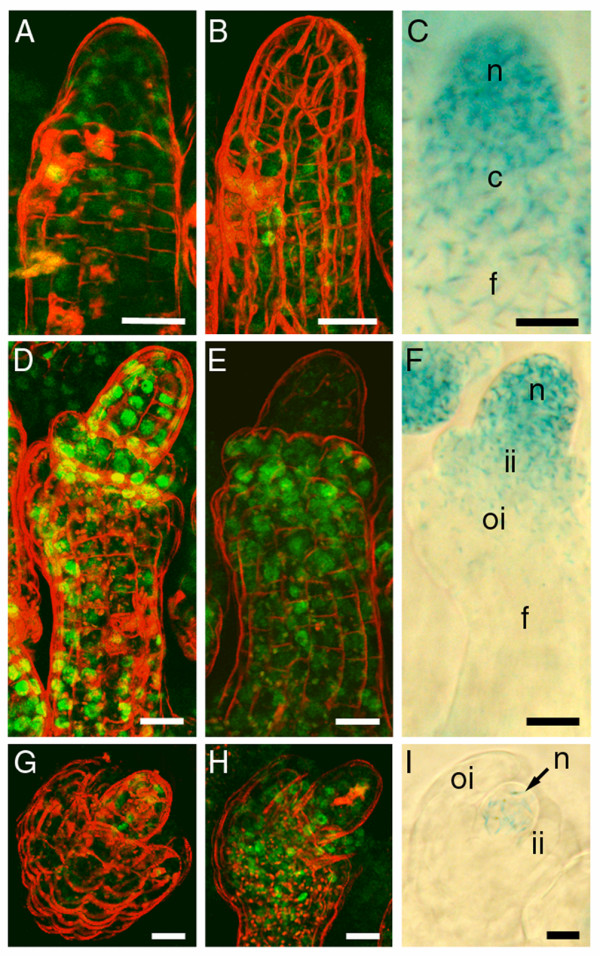
**Localisation of SEP3:GFP, AG:GFP and expression of *pWUS:GUS *during ovule development**. Confocal microscopic analysis of SEP3:GFP localisation (A, D, and G) and AG:GFP localisation (B, E, and H) during ovule development. GFP tagged MADS domain proteins are detected as green signal and cell membranes are stained with the red dye FM4-64. DIC microscopy of *pWUS:GUS *expression in developing ovules (C, F, and I) where GUS activity is detected as a blue colour. (A) SEP3:GFP signal in the nucellus, chalaza, and funiculus of an ovule at stage 2-II. (B) AG:GFP signal in the chalaza and funiculus of an ovule at stage 2-II. (C) *pWUS:GUS *expression in an ovule at stage 2-II in an increasing basal-to-apical gradient. (D) High SEP3:GFP signal specifically in the nucellus, the inner integument, and the funiculus in an ovule at stage 2-III. (E) AG:GFP signal in an ovule at stage 2-III, with beginning signal in the nucellus and the highest signal in the developing inner and outer integuments. (F) *pWUS:GUS *expression in the nucellus and the two integuments of an ovule at stage 2-III in an increasing basal-to-apical gradient. (G) SEP3:GFP signal in an ovule at stage 2-IV with the highest signal in the nucellus. (H) AG:GFP signal in the nucellus, the two integuments, and the funiculus of an ovule at stage 2-IV. (I) *pWUS:GUS *expression only in the nucellus in an ovule at stage 2-IV. (c) chalaza; (f) funiculus; (ii) inner integument; (oi) outer integument; (n) nucellus. All scale bars 10 μm.

Both SEP3:GFP and AG:GFP were present from the protrusion stage of ovule development stage 1 onwards (stages after [50]). At the beginning of stage 2 SEP3:GFP was present in the whole protrusion, while AG:GFP seemed to be limited to the funiculus and the chalaza with hardly any fluorescence in the nucellus (Figures 7A and 7B). During the initiation of the inner and the outer integuments, stages 2-II to 2-III, the amount of AG:GFP protein in the nucellus increased. At the same time, the initiating inner and outer integuments showed the highest AG:GFP levels (Figure 7E). Also SEP3:GFP signal peaked during the stages 2-II to 2-III, with the highest signal in the initiating inner integument and the nucellus (Figure 7D). After stage 2-III, both AG:GFP and SEP3:GFP were clearly present in the nucellus (Figures 7G and 7H). These spatio-temporal patterns of AG:GFP and SEP:GFP fit with the reported *AG *mRNA expression [51] and the *SEP3 *mRNA expression [20]. However, no *AG *expression was seen in the nucellus [51], whereas we did see increasing AG:GFP signal in the nucellus (Figures 7E and 7H). This could be the result of AG:GFP transport from the developing integuments towards the nucellus. The transcriptional *pWUS*:*GUS *reporter line [43] showed that *WUS *expression gradually becomes restricted to the integument primordia and the nucellus in the stages 2-II to 2-III (Figures 7C and 7F), until it was only present in the nucellus after stage 2-III (Figure 7I). *WUS *expression peaked in the nucellus during the stages 2-II to 2-III and afterwards diminished. Therefore it seems that this proximal-distal down-regulation of *WUS *expression in the different ovular tissues is preceded by increasing proximal-distal AG:GFP and SEP3:GFP signals and only occurs in the tissues where both SEP3:GFP and AG:GFP were present, supporting the hypothesis that SEP3 and AG are together involved in the repression of *WUS *expression [49].

## Conclusion

The results described here show that in some cases a discrepancy exists between the previously reported mRNA expression patterns and our protein localisations. Therefore, mRNA expression patterns alone are not sufficient to form hypotheses about gene function and they need to be supported by protein localisation data. For instance, in a recent paper [52] it was suggested that *FUL*, unlike *AP1*, cannot be directly involved in the regulation of flowering time genes (e.g. *SUPPRESSOR OF OVEREXPRESSION OF CONSTANS1*), simply because it is not expressed in the early flower bud stages. Our localisation data, however, shows that FUL:GFP is present in the early flower bud stages and therefore could have the same function as AP1 in regulating these flowering time genes. This is also in agreement with the hypothesis that both AP1 and FUL act as hubs between the flower induction protein network and the floral organ protein network [18], and previous genetic studies that showed redundancy between AP1 and FUL during the floral transition [9]. Other examples, such as the asymmetric localisation of SEP3 in the epidermis of both sepals and petals and its possible role in the adaxial/abaxial patterning of these organs, the presence of FUL protein in the replum of developing pistils, or the presence of AG in the nucellus of the developing ovule during the down-regulation of *WUS *were also not apparent from the reported mRNA patterns. These cases demonstrate the importance of studying protein patterns and protein levels for a better understanding of transcription factor functioning. Some caution is needed however, as we analysed the behaviour of tagged proteins, and we cannot exclude that the localisation patterns of these fusion proteins differ from those of the native proteins. It is possible that the increased size of the fusion protein could have an effect on the transport abilities of the protein, or that the presence of the tag could interfere with the ability to form (multimeric) protein complexes. Furthermore, the presence of the tag could change the stability of the protein, which could lead to a different localisation pattern. We also have to realise that the transgenes are inserted on other positions in the genome than the endogenous genes, which may cause some positional effects on expression pattern and level. Nevertheless, because of the capacity of the AG, FUL and AP1 fusion proteins to complement their respective mutants and the lack of ectopic expression phenotypes in the transgenic lines during floral development, it is very likely that the localisations of the fusion proteins mimic the patterns of the endogenous proteins.

Discrepancies between mRNA and protein patterns may suggest non-cell autonomous action of the proteins by intercellular transport. This has been shown for a number of transcription factors, such as KNOTTED-1 in maize [53], and SHORTROOT [54] and CAPRICE [55] in *Arabidopsis*. Also MADS domain proteins may move from one cell to another, as has been reported for the *Antirrhinum majus *MADS domain proteins DEFICIENS and GLOBOSA [23]. In our study there are a few examples where the presence of MADS domain proteins could be due to intercellular transport. For instance, the low level, but distinct presence of SEP3 in the epidermis of the inflorescence meristem and stage 1 flower buds could be due to epidermal transport from high expressing stage 2/3 flower buds. Another example is the increasing AG presence in the nucellus in the developing ovule, where the high expressing integuments might be the source of the AG proteins in the nucellus. Also, as previously suggested [22], the FUL proteins in the replum in developing pistils could originate from the high expressing valve cells.

Subcellular localisations of proteins can provide clues on the regulation and functioning of transcription factors. The subcellular SEP3:GFP localisation in the cytoplasm in the area of the floral meristem that will become whorl 2 and 3 is an example of a localisation that is not expected based on the nature of transcription factors. The cytoplasmic localisation could indicate that the SEP3:GFP protein in the cytoplasm is not in a dimeric form, and therefore cannot be transported to the nucleus [11-13,24]. Alternatively, it could also be that post-translational modifications cause the cytoplasmic retention of the SEP3:GFP protein, possibly facilitating intercellular transport [56] or breakdown of the protein [57]. Recently, it has also been shown that the MADS domain protein MPF2 from *Physalis floridana *is located in the cytoplasm and imported into the nucleus upon hormone treatment with cytokinin [58]. This shows that non-nuclear localisation is an intriguing mechanism for the regulation of transcription factor functioning.

Summarising, our analysis with GFP tagged proteins expressed under the control of the endogenous promoter revealed the spatio-temporal dynamics of the MADS domain proteins in various tissues of the living plant, leading to a deeper understanding of the behaviour of these MADS domain proteins and allowing the formation of new hypotheses about their function and regulation during early and later stages of floral development.

## Methods

### *Arabidopsis thaliana *plants

All plants were grown at 22°C in growth chambers under a long-day light regime (16 h light/8 h dark).

The construction of transgenic lines expressing g*AG:GFP*, g*FUL:GFP *and g*SEP3:GFP *was previously described [29]. The *AG *genomic clone has a promoter region of approximately 2.6 kb upstream from the translational start, the *FUL *genomic clone has a promoter region of approximately 2 kb and the *SEP3 *clone has a promoter region of approximately 1.5 kb. To make the translational g*AP1*:*GFP *fusion construct, a genomic clone fragment of *AP1 *(6616 bp) was amplified with the following two gene specific primers: the forward primer PDS298 (5'-GGGGACAAGTTTGTACAAAAAAGCAGGCTGTTTAACATCCAAGATTTGTTTTACATAATCGTTAC-3') located 2992 bp upstream from the translational start, and the reverse primer PDS297 (5'-GGGGACCACTTTGTACAAGAAAGCTGGGTCTGCGGCGAAGCAGCCAAGGTT-3') lacking the stop codon at the 3' end of the coding sequence. The amplified product was inserted into the pENTR/D-TOPO vector (Invitrogen) and, after sequence controls, recombined into the binary vector pMDC107 [59]. *Arabidopsis *plants were transformed with *Agrobacterium tumefaciens *strain GV3101 using the floral dip method [60]. Transformed seeds were selected on LB agar plates with 10 μg/ml hygromycin, and stable transgenic lines were maintained afterwards. Furthermore, a transcriptional *pWUSCHEL*:*GUS *line [43] was used to analyse the expression pattern of *WUSCHEL *in developing ovules.

### Complementation experiments

Stable transgenic plant lines expressing *gAG:GFP *and *gFUL:GFP *in wild type Col-0 background were crossed with the SALK_014999 *ag *T-DNA insertion mutant line and the *ful-1 *mutant [22] respectively, while the *gAP1:GFP *construct was directly transformed into the SALK_056708 *ap1 *T-DNA insertion mutant line. In the progeny, the presence of the wild type allele, the mutant allele, and the GFP tagged MADS box gene construct was determined by PCR and CLSM. For *AG *the following primer pairs were used: *AG *wild type allele, forward primer PRO182 (5'-GGATCCATGGCGTACCAATCGGAGCT-3') annealing immediately after the START codon and reverse primer PDS1985 (5'-CATTTCCTTCAGCCTATATTACC-3') located in the 3' UTR 18 bp downstream of the STOP codon; *ag *T-DNA mutant allele, forward primer PRO433 (5'-CACCGATCAAAGACTACACATCAC-3') located in the 5' UTR 2634 bp upstream of the START codon and reverse primer PDS404 (5'-TGGTTCACGTAGTGGGCCATCG-3') located on the left border of the T-DNA. The presence of *gAG:GFP *was determined by CLSM in the inflorescences. For *FUL*, the following primer pairs were used: *FUL *wild type allele, forward primer PDS1024 (5'-CTTACGTCACTGTAGACTCACG-3') located in the 5' UTR 201 bp upstream of the START codon and reverse primer PDS1023 (5'-AAAGAGTGAGATAGTTCTACTCG-3') in the 3' UTR 16 bp downstream of the STOP codon; *ful-1 *mutant allele, forward primer PDS1025 (5'-TTCATCCCTTTTTCAGGGTTGTC-3') corresponding with the inserted DsE element and reverse primer PDS1023; and g*FUL:GFP*, forward primer PDS920 (5'-ATCACTTACGTCACTGTAGACTCACG-3') in the 5' UTR 204 bp upstream and reverse primer PDS914 (5'-CATCATGTTTGTATAGTTCATCCATGCC-3') 5 bp upstream of the STOP codon of m*GFP6*. For *AP1*, the following primer pairs were used: *AP1 *wild type allele, forward primer PDS912 (5'-AAAACTTTAGGGCCGTAGTGAAGTGAAC-3') 385 bp downstream of the START codon and reverse primer PDS1105 (5'-ATTGGATGAAAAGAGCCTAGCCAC-3') in the 3' UTR 89 bp downstream of the STOP codon (which will give no product for the *ap1 *T-DNA mutant allele); g*AP1:GFP*, forward primer PDS912 and reverse primer PDS915 (5'-GACCAGGGTTGGCCATGGAACAGG-3') 183 bp downstream of the START codon of *mGFP6*.

### Confocal laser scanning microscopy

To observe the localisation of the GFP tagged proteins in living plant tissue, inflorescence material was dissected until the relevant meristems and flower buds became visible. After stage 5 flower buds, when sepals started to enclose the floral meristem, it became dificult to visualise the underlying developing floral organs without dissecting the flower buds. The tissues were embedded as previously described [29]. The dye FM4-64 (Molecular Probes, Leiden, The Netherlands) was used as a red counter stain for cell membranes and added at a concentration of 5 μM to the embedding mixture of 0.8% agar, 0.5 × MS. The incubation time of the sample in the embedding mixture with FM4-64 was at least 20 minutes. Confocal Laser Scanning Microscopy (CLSM) of the living plant tissue was performed with a Zeiss LSM 510 inverted confocal microscope using a 40 × C-Apochromat (NA 1.2 W korr) lens. Both GFP and the FM4-64 dye were excited with the 488 nm line of an Argon ion laser. The GFP emission was filtered with a 505–530 nm band pass filter, while the FM4-64 dye emission and red autofluorescence was filtered with a 650 nm long pass filter. The optical slices in the confocal z-stacks were made as a sum of 4 scans and were median filtered afterwards. Three-dimensional projections of the obtained confocal z-stacks were made with the Zeiss LSM Image Browser version 4 and adjusted with Adobe Photoshop version 5.0.

### GUS assay

To analyse the expression pattern of *WUSCHEL *in developing ovules, inflorescences of the *pWUS*:*GUS *line were fixed and a β-glucuronidase (GUS) assay was performed overnight at 37°C as previously described [61] (modified from [62]). After GUS detection and chlorophyll removal, the inflorescences were kept in Hoyer's solution (7.5 g Arabic gum, 100 g chloral hydrate, 5 ml glycerol, and 60 ml water). Whole siliques of one inflorescence were put under a cover slip and observed with a Nikon Optiphot microscope. Bright field images of the ovules were taken with a Leica DFC320 digital camera using a 40× Plan DIC objective.

## Authors' contributions

For the MADS domain protein localisations SdF, AV and KK created the GFP-tagged MADS box constructs, and SU selected the transgenic plant lines and analysed the protein localisation patterns with CLSM. For the *WUS *expression pattern in ovules RI performed the GUS assay on inflorescences of *pWUS:GUS *plants and SU imaged the ovules. SU wrote the manuscript and RI and GA critically revised it. All authors read and approved the final manuscript.
